# Production and Characterization of Superhydrophobic and Antibacterial Coated Fabrics Utilizing ZnO Nanocatalyst

**DOI:** 10.1038/s41598-018-22324-7

**Published:** 2018-03-02

**Authors:** Mohamed Shaban, Fatma Mohamed, Semsem Abdallah

**Affiliations:** 1 Nanophotonics and Applications (NPA) Lab, Department of Physics, Faculty of Science, Beni -Suef University, Beni-Suef, 62514 Egypt; 2 Department of Chemistry, Faculty of Science, Beni-Suef University, Beni-Suef, 62111 Egypt

## Abstract

Dirt and microorganisms are the major problems in textiles which can generate unpleasant odor during their growth. Here, zinc oxide (ZnO) nanoparticles prepared by sol-gel method were loaded on the cotton fabrics using spin coating technique to enhance their antimicrobial properties and water repellency. The effects of ZnO precursor concentration, precursor solution pH, number of coating runs, and Mg doping percent on the structures, morphologies, and water contact angles (WCA) of the ZnO-coated fabrics were addressed. At 0.5 M concentration and pH7, more homogeneous and smaller ZnO nanoparticles were grown along the preferred (0 0 2) direction and uniformly distributed on the fabric with a crystallite size 17.98 nm and dislocation density 3.09 × 10^−3^ dislocation/nm^2^. The substitution of Zn^**2+**^ with Mg^**2+**^ ions slightly shifted the (002) peak position to a higher angle. Also, the zeta potential and particle size distribution were measured for ZnO nanoparticle suspension. A superhydrophobic WCA = 154° was measured for the fabric that coated at 0.5 M precursor solution, pH 7, 20 runs and 0% Mg doping. Moreover, the antibacterial activities of the ZnO-coated fabric were investigated against some gram-positive and gram-negative bacteria such as *Salmonella typhimurium*, *Klebsiella pneumonia*, *Escherichia coli*, and *Bacillus subtilis*.

## Introduction

For textile industry, there are growing attempts to develop textile materials with more proper properties. Textiles or fabrics are important in other industries such as agricultural industry, transportation, building material and healthcare industry. Cotton fabrics are widely used in our daily life because of their excellent properties such as softness, affinity to skin, bio-degradability and regeneration property^1,2^. The abundant water-absorbing hydroxyl groups on the cotton surface make the fiber absorbent and easily stained by the liquids. The stains and liquids lead to the growth of different types of bacteria. The microbial infectivity has a bad effect on health care and food industry. This increases the awareness of consumers toward health and hygiene. Therefore, the need for self-cleaning and antibacterial textiles is an essential requirement for our daily life.

In the recent decade, nanotechnology has a clear role in processing and finishing textile. Nanostructured materials are attracting in the textile field because of their enormous surface area, unique physical and chemical properties compared to bulk materials. Hence, they are suitable for preparing clean surfaces that can be used in various applications^3,4^. Different hygienic nanoparticles were loaded on the fabrics due to their unique properties in decomposing undesirable contaminants^5–8^. These nanostructures have a dual effect on reducing bacterial colonization on textile surfaces and for introducing self-cleaning properties to the fibers. The self-cleaning feature is based on the Lotus effect (superhydrophobic property) or the photo superhydrophilic property^9,10^. The design of superhydrophobic fabrics via some different materials to make water-repellent and clean textiles was the scope of attention for many Scientists^3,4,11–15^. According to these studies, the wettability of the surface is mainly dependent on its chemical composition and the geometrical structure of the surface. Many studies were done by using different coatings as Pt, Au and Ag NPs for obtaining a good self-cleaning and antimicrobial textile. However, they have drawbacks such as high cost of coating materials and complex coating techniques such as plasma treatment and RF sputtering^3,16^.

Some metal oxide nanoparticles (NP) are receiving increased attention as self-cleaning or/and antimicrobial coatings because of their stability in aqueous solutions, ability to reduce bacterial colonization, longer lifetimes, and chemical stability in high pressure or temperature^14^. Zinc oxide (ZnO) is a semiconductor material that can be tuned in different nanostructure morphologies^17–20^. ZnO has many significant features such as chemical and physical stability, unique optical properties, high catalysis activity and effective antibacterial activity^17–22^. In addition to its low cost and high photochemical reactivity, ZnO has a wide band gap of 3.37 eV with a large excitation binding energy of 60 meV^1^. Also, ZnO is bio-safe and biocompatible for medical applications as effective antimicrobial agents, drug carriers, and bio-imaging probes^8,15,16,23,24^. Consequently, many attempts have been carried out to impregnated ZnO nanoparticles onto the cotton fiber through two general routes for the development of low-cost, antibacterial and self-cleaning textiles. The first route was to coat the prepared ZnO nanocrystals onto cotton fibers simply by cure process^25^. The second route has been carried out using the ultrasonic irradiation as an efficient method for coating nanomaterials onto the surface of cotton fibers and other substrates^26,27^.

Although there are many studies in the literature regarding the antibacterial properties of ZnO NPS, the mechanism of their antibacterial behavior is still not completely understood. Several authors have suggested an antibacterial action as consequence of the action of reactive oxygen species (ROS) that are formed on the ZnO NPS surface by a photocatalytic process^8^. Also, there are strong demands for simple, low-cost and massive product techniques for the homogeneous and controlled loading of ZnO nanostructures on the cotton fabrics. Also, the coating parameters and properties of deposited nanostructures must be optimized to produce cotton fabrics with superhydrophobic and enhanced antibacterial activities for industrial applications.

This study aims to fabricate ZnO NPs- modified fiber by spin coating technique to obtain a hydrophobic fiber with super contact angle and excellent antibacterial activities under non-photocatalytic reaction. The sol-gel spin coating technique is applied to modify the surface of the textile because it offers far-reaching possibilities for creating new surface properties, homogeneity of the obtained coatings, excellent control of the stoichiometry, large area substrate coating, and the ability to scale up to industrial fabrication^28^. This study investigated the optimum conditions for incorporating ZnO NPs and effects of precursor concentration, pH value, a number of coating runs, and Mg –doping ratio on structures, morphologies, and WCA of the coated fabrics. The antibacterial activities of the optimized sample have been investigated for some species of gram-positive and gram-negative bacteria.

## Results and Discussion

### Morphological Properties

#### Effect of precursor concentration

**Figure 1:**
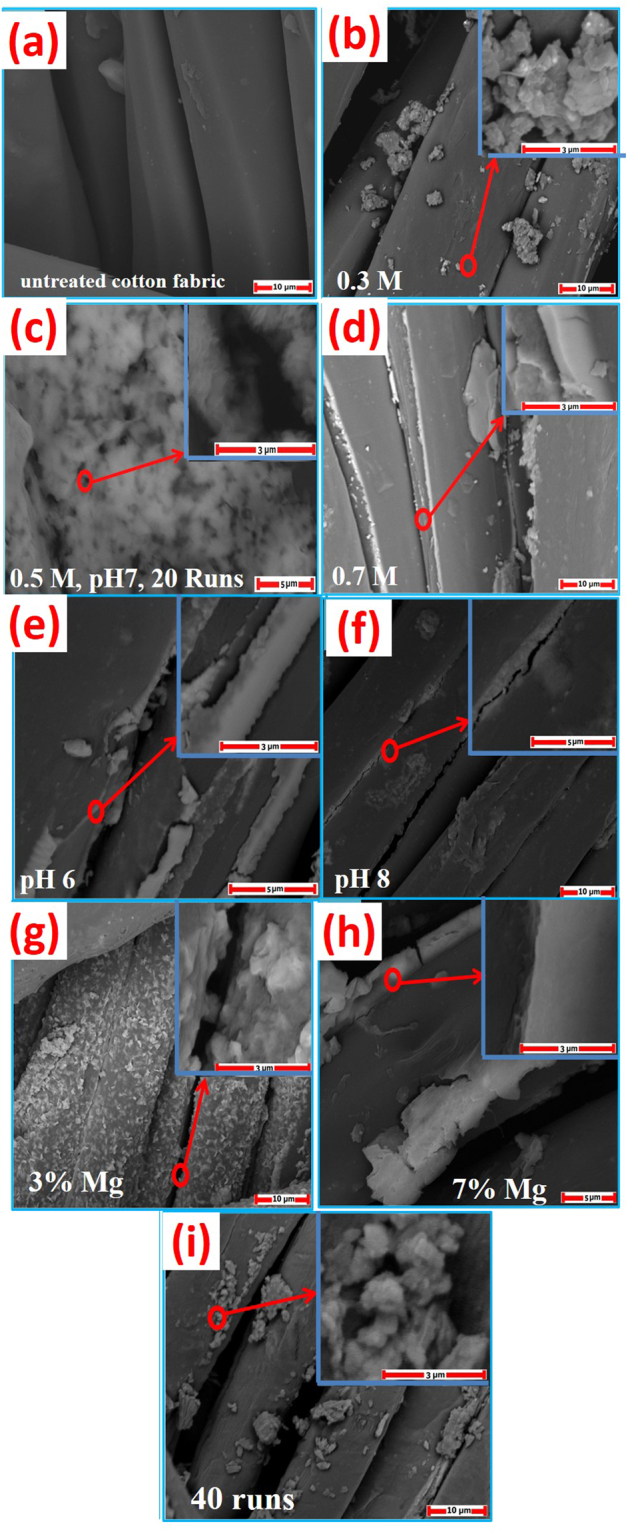
SEM images of (**a**) untreated cotton fabric; ZnO coated fabric at different concentrations of ZnO precursor solution: (**b**) 0.3 M, (**c**) 0.5 M and (**d**) 0.7 M at pH 7 and 20 runs; at different pH values: (**e**) pH 6 and (**f**) pH 8 at 0.5 M precursor concentration and 20 runs; at different Mg doping percent: (**g**) 3% Mg and (**h**) 7% Mg at 0.5 M precursor concentration, pH 7 and 20 runs; and (**i**) at 40 coating runs using 0.5 M precursor concentration, pH 7 and 0% Mg.

Figures 1 and S1(supplementary data) show SEM images of the uncoated fabric and the ZnO-coated fabrics at different conditions. Figure S1(a) shows SEM image of ZnO nanoparticles with non-spherical morphology. The uncoated fabric showed a smooth surface with no particles on its surface, Figs 1(a) and 1S(b). Figure 1(b,c) showed SEM images of coated fabrics using different ZnO precursor concentrations (0.3, 0.5 and 0.7 M). After the incorporation of 0.3 M ZnO nanoparticles, Fig. 1(b), it was found that the loading of fabric was non-homogeneously with agglomerated ZnO nanoparticles. ZnO was not attached very well to the fabric, and no particles were formed between fibrils. The fabric was not entirely coated with ZnO nanostructures. This is because of the low concentration of the solution. Then, the active sites of ZnO are limited, and this weakens the hydrogen bonds that can be formed between the fabric and ZnO nanostructures. As a result, ZnO nano agglomerates are only loaded in some places on the fabric as shown in the inset.

At 0.5 M concentration, more homogeneous ZnO nanostructures were formed on the fabric as seen in Fig. 1(c). The deposited nanostructures are self-assembly to cover the surface and non-uniform nanoporous features are observed as seen in Fig. S1(c). Excellent adhesion between ZnO and the fabric was observed because the oxygen vacancies and the active sites of ZnO increased due to increasing of their concentration^29^. It can also be seen that the fabric is entirely coated with ZnO nanostructures. The fabric was completely covered with layers of ZnO at 0.7 M concentration, Figs 1(d) and S1(d). These layers produce a shield on the cotton fabric besides formation of ZnO agglomerations on the fabric surface due to the high concentration of the precursor.

#### Effect of pH

The pH effect on the morphology was studied at pH 6, 7 and 8. Changing the pH of the solution to more acidic results in non-uniformity of ZnO layer and low adhesion to the fabric. Non-uniform significant agglomerations are formed at pH 6, Figs 1(e) and S1(e). This agglomeration is a result of the acidic pH of the Zn(OH)_2_ sol during the synthesis process. That is attributed to the lack sufficient of OH^−^ ions presented in the sol which allow the nucleation and growth of ZnO and the formation of particles^30^. Figure 1(c) shows a homogeneous distribution of ZnO nanostructures when the pH is increased to pH 7 values. At more alkaline solution (pH 8), OH^−^ ions concentration increased, and this resulted in the growth of ZnO crystallites to form a thick ZnO coating to the fabric, as seen in Figs 1(f) and S1(f).

#### Effect of Mg doping percent

Doping with specific elements is a facial method that could be used to improve the physical and chemical properties of ZnO material for some particular needs or applications. Mg is expected to make a good solid solution with ZnO because of the wider bandgap of MgO^31^. So, it is important to show the effect of Mg-doping ratio on the morphological, structural, and hydrophobic properties of Mg-doped ZnO-coated fabrics. We have chosen the Mg-doped ZnO system in the present study because Mg^2+^ ions are readily incorporated into the ZnO lattices by substitution since the radius of Mg^2+^ (0.72 Å) is closer to that of Zn^2+^ (0.74 Å)^32^. Figure 1(g,h) shows the effect of Mg doping % on the morphology of ZnO-coated fabrics. This figure is indicating that the morphology of ZnO film is significantly affected by Mg doping ratio^33,34^. Also, the thickness of the coated layer on the fabric surface increased with the incorporation of Mg^2+^ions from 3% (Figs 1(g) and S1(g)) to 7% (Figs 1(h) and S1(h)). It is observed from Fig. 1(h) that, raising the Mg doping ratio to 7% is resulted in a much smoother and thicker layer by the agglomeration of the nanoparticles on the fabric surface.

#### Effect of number of runs

The other factor affecting the surface of fabrics is the number of coating runs. With increasing the number of runs, the thickness of the ZnO layer increased to coat the entire fibril at 20 runs, as shown in Fig. 1(c). Increasing the number of runs to 40 runs resulted in the agglomeration of ZnO nanoparticles on the fabric surface, as shown in Fig. 1(i).

### Structural studies

**Figure 2:**
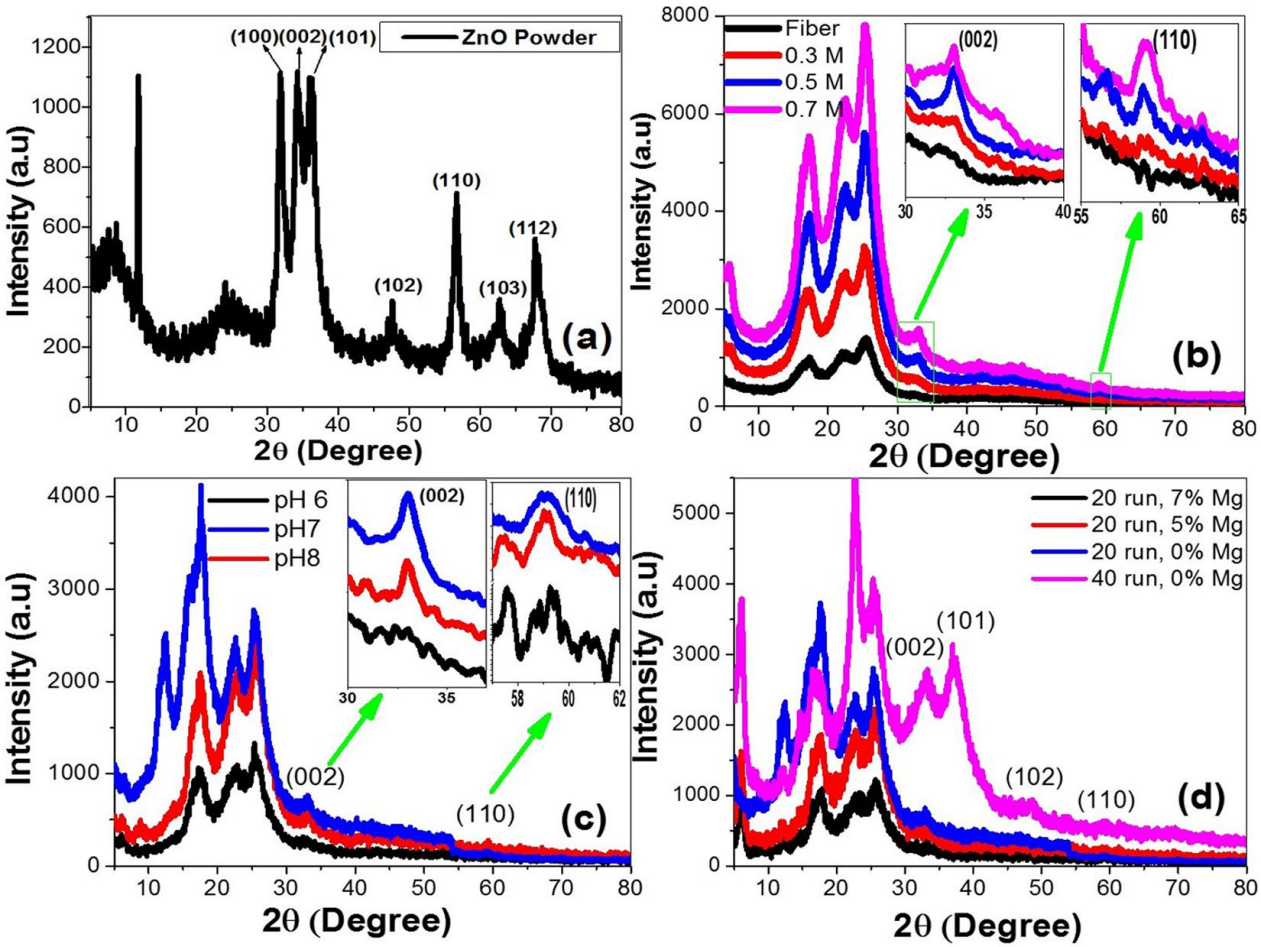
XRD patterns of (**a**) ZnO nanoparticles prepared by the sol-gel method at 0.5 M precursor concentration, pH 7 and 0% Mg doping percent; ZnO-coated fabric at different (**b**) concentrations of ZnO precursor solution, (**c**) pH values, and (**d**) coating runs and Mg doping percent.

XRD analysis can provide information about the crystal structure and chemical composition of the samples. Figure 2(a) shows the XRD pattern of ZnO nanostructured powder annealed in air at 150 °C for 2 h. The peaks at 2θ = 31.8, 34.2, 36.2, 47.6, 56.7, 62.7 and 67.99° of ZnO nanoparticles are corresponding to the growth of ZnO nanocrystallites along (1 0 0), (0 0 2), (1 0 1), (1 0 2), (1 1 0), (1 0 3) and (1 1 2) orientations (JCPDS No. 01-078-4603). There was a shift of 2θ by approximately −0.3° because of the low annealing temperature^35,36^. The peaks at 2θ = 7.6, 11.8 and 24.1° are due to layered hydroxide zinc acetate (LHZA), which is formed as an intermediate during the formation of ZnO. The preferred orientation for the growth of LHZA crystallites is (0 0 1) plane at 2θ = 11.8°. These LHZA crystallites didn’t convert to ZnO because the annealing temperature is lower than the temperature that required for the complete conversion to ZnO^37–39^.

The average crystal size was calculated from Debye–Scherer relation; D = 0.94λ/β cosθ, where λ is the X-ray wavelength, β is the line broadening at the half maximum intensity (FWHM) in radians, and θ is the Bragg angle. The calculated D values are presented in Table 1, and the average crystal size of the synthesized ZnO nanoparticle is 13.4 nm.

**Table 1:** The XRD data for the ZnO NPs and ZnO-loaded fabrics at different conditions, pH values, the number of runs, and Mg%; the texture coefficient (TC), the crystallite size (D), and the dislocation density (δ).

	Planes	D (nm)	δ (10^−3^ dislocation/ m^2^)	TC
ZnO	(1 0 0)	15.86	3.98	1.674
	(0 0 2)	13.66	5.36	1.817
	(1 0 1)	12.00	6.94	1.742
	(1 0 2)	8.29	15.55	0.315
	(1 1 0)	20.86	2.30	1.146
	(1 0 3)	8.89	12.65	0.405
	(1 1 2)	13.77	5.27	0.781
**Effect of concentration**				
0.3 M	(0 0 2)	20.3	2.43	1
0.5 M	(0 0 2)	17.98	3.09	1.656
	(1 1 0)	13.99	5.10	0.344
0.7 M	(0 0 2)	21.01	2.30	1
**Effect of pH**				
pH 6	—	—	—	—
pH 7	(0 0 2)	17.98	3.09	1.656
	(1 1 0)	13.99	5.10	0.344
pH 8	(0 0 2)	13.24	5.70	1
**Effect of number of runs**				
20 run	(0 0 2)	17.98	3.09	1.656
	(1 1 0)	13.99	5.10	0.344
40 run	(0 0 2)	5.66	31.22	1
**Effect of Mg doping percent**				
0% Mg	(0 0 2)	17.98	3.09	1.656
	(1 1 0)	13.99	5.1	0.344
5% Mg	(0 0 2)	7.92	15.94	1.588
	(1 0 2)	1.29	600.93	0.412
7%Mg	(0 0 2)	13.24	5.70	1

The preferred orientations of ZnO nanostructured powder are evaluated by the texture coefficient (TC) of the *hkl* planes. TC (*hkl*) is calculated from the X-ray data using the well-known formula^19,20^;

1 $$TC(hkl)=\,\frac{I(hkl)/{I}_{o}(hkl)}{\frac{1}{N}{\sum }^{}I(hkl)/{I}_{o}(hkl)},$$

where *I* refer to the measured or normalized intensity, *I*_*o*_ the corresponding standard intensity given in JCPDS data, *N* is the number of reflections^40,41^. TCs values for the planes of ZnO nanostructured powder and ZnO coated fabric at different conditions are calculated, and the obtained values are listed in Table 1. It is observed from Table 1 that TC (002) > TC(101) > TC(100) > 1. Then, ZnO nanoparticles show more preferred orientation along (0 0 2) plane.

This designates a preferential growth along the c-axis, which is related to the minimum value of the (002) surface free energy (9.9 eV/nm^2^) in the wurtzite ZnO structure^42^. This c-axis preferred orientation be critical for ultrasonic oscillators and transducer devices^18^. The dislocation density(δ), which represents the number of defects in the crystal, is calculated using the Williamson–Hall equation, δ = 1/D^2^ ^42,43^. The dislocation density is inversely proportional to D^2^ and its values are listed in Table 1.

Figure 2(b) illustrates the XRD patterns of cotton fabric and ZnO loaded-cotton fabrics synthesized by different ZnO precursor concentrations (0.3, 0.5, 0.7 M). The peaks at 2θ = 17.4°, 22.3°, and 25.2° are the diffractive peaks of cotton fiber^44^. At different concentrations, the preferred (0 0 2) peak of ZnO appears at all concentrations in addition to the peak (1 1 0) which appears only at 0.5 M and 0.7 M as seen in the insets of Fig. 2(b). This confirms the growth of hexagonal phase ZnO on cotton fabric. No additional metallic Zn or impurity phases are detected, which indicates the loading of pure crystalline ZnO NPs on the cotton fabrics and the precursors have been fully transformed into ZnO phase. Disappearing of the other peaks of ZnO nanoparticles may be attributed to the loading of a small amount of ZnO nanoparticles on the cotton^44^. The intensity of the (0 0 2) peak increases with increasing the precursor concentration. Also, the peaks intensity of the cotton fabric increased with increasing the precursor concentration. ZnO shows preferred orientation along (0 0 2) direction. Increasing the concentration of ZnO from 0.3 M to 0.5 M results in increasing the texture coefficient of the preferred growth direction (0 0 2) as listed in Table 1. The texture coefficient decreased when the concentration rose to 0.7 M. It was observed from Table 1 that the crystallite size in (0 0 2) orientation is reduced from 20.3 nm to 17.98 by increasing the precursor concentration from 0.3 M to 0.5 M, then rose to 21.01 nm when the concentration reached 0.7 M due to the agglomeration of ZnO nanoparticles. With increasing the precursor concentration up to 0.5 M, the value of D decreases and hence the value of δ increases to 3.09 × 10^−3^ dislocation/nm^2^ (0 0 2) orientation. This increase of δ gives rise to disorders and crystal defects in the ZnO lattice.

Figure 2(c) shows the XRD patterns of the fabrics loaded by ZnO at different pH values. For the prepared sample at pH 6 (acidic medium), there aren’t characteristic peaks for ZnO. ZnO structure cannot be synthesized at pH 6 because of the high concentration of H^+^ ions and the small concentration of OH^−^ ions in the sol^30^. For the neutral condition at pH 7, the number of H^+^ ions and OH^−^ are equivalent. As a result, the characteristic peaks of ZnO were observed for the orientations (002) and (110), insets of Fig. 2(c).

At pH 8 (alkaline medium), a sufficient amount of OH^−^ is available to form ZnO at the preferred orientation (0 0 2)^30,45^. At pH 7, the (0 0 2) and (110) peaks are sharper and stronger, insets of Fig. 2(c), that confirmed the better crystallization and loading of ZnO on the cotton fabric. From Table 1, the crystallite size for (0 0 2) plane decreased from 17.98 nm to 13.24 nm as the pH increased from 7 to 8^46^. Also, the dislocation density δ increased with increasing the pH value as listed in Table 1.

Figure 2(D) shows XRD patterns of cotton fabrics coated with Mg-doped ZnO nanoparticles, whereas the Mg doping ratio was changed from 0% to 7%. Doping with Mg did not cause any additional diffraction peaks with ZnO peaks because Mg^**2+**^ ions were substituted into Zn^**2+**^ ion sites or incorporated into interstitial sites in the ZnO lattice^40^. It was observed that the growth of Mg-doped ZnO is in the (0 0 2) crystal plane and its intensity increases with increasing Mg doping percent. This confirms the substitution of Zn^**2+**^ ions with Mg^**2+**^ ions. Because the ionic radius of Mg^2+^ ion is smaller than the ionic radii of Zn^2+^, the (002) peak position was slightly shifted to a higher angle by increasing the Mg percent. As the doping percent reached 5% Mg, an additional peak appeared which corresponding to (1 0 2) crystal plane of ZnO. This peak disappeared when the Mg doping percent increased to 7%. It can be observed from Table 1 that the texture coefficient of (0 0 2) plane decreased with increasing the Mg doping percent. The dislocation density increased with increasing the Mg-doping percent to 5% then decreased after increasing the Mg-doping percent to 7%.

Also, Fig. 2(d) shows XRD patterns of cotton fabrics loaded with ZnO nanoparticles at two different number of coating runs; 20 and 40 runs. Both samples show (0 0 2) diffraction peak and grow preferentially along c-axis perpendicular to the substrate surface. For the sample that fabricated at 20 runs, there is another peak corresponds to (1 1 0) orientation plane of ZnO. By decreasing the number of runs, the crystallite size of the loaded ZnO is increased from 5.7 to 17.98 nm as shown in Table 1. Also, the dislocation density was increased from 3.09 × 10^−3^ to 31.22 × 10^−3^ dislocation/nm^2^ by increasing the number of runs due to the agglomeration of ZnO nanoparticles.

From the previous results, the proper precursor concentration, number of runs, pH value, and Mg-doping ratio can significantly improve the crystalline quality of ZnO-coated fabrics. This may be interpreted regarding the decrease in surface energy as the ZnO atoms are trapped at or migrate down the grain boundaries^18,47^.

### FTIR-spectroscopy for ZnO - coated fabrics

**Figure 3:**
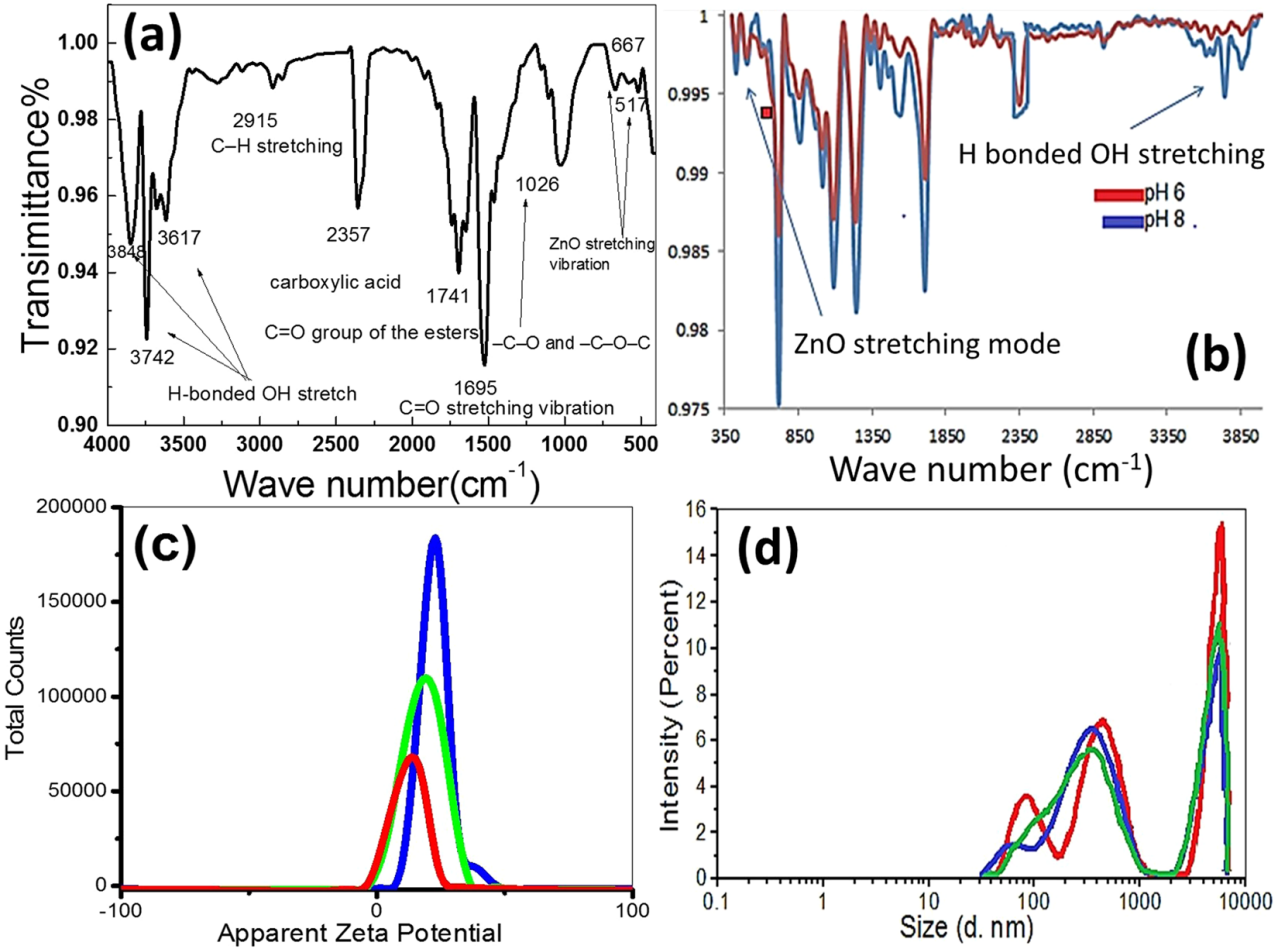
(**a**) The FT-IR spectrum of ZnO-coated fabric at the optimized conditions, (**b**) FTIR spectra of ZnO-coated fabrics at pH 6 and pH8, (**c**) zeta potential, and (**d**) particle size distribution of ZnO nanoparticle suspension.

The FT-IR spectrum of ZnO-coated cotton fabric was measured in the range between 400–4000 cm^−1^ and presented in Fig. 3(a). The absorption bands in the region 3600–3900 cm^−1^ were attributed to stretching vibration modes of OH groups of cotton fabrics. The band at 2915 cm^−1^ is determined by υ(–C-H) stretching vibration. Also, there is an absorption band at 2357 cm^−1^ of O-H stretching, which ascribed to carboxylic acid due to oxidation of primary hydroxyl sites. The peaks located at 1741 and 1695 cm^−1^ are assigned to the C=O stretching vibration of cellulose. The absorption band at 1062 cm^−1^ is ascribed to -C-O and –C-O-C stretching modes. The bands at around 667, 580 and 517 cm^−1^ can be attributed the Zn-O vibrational modes^48^. Figure 3(b) shows FTIR spectra of two samples coated at pH 6 and pH 8. It can be seen that as the pH increased, stronger vibrational modes are observed. Also, this figure clearly indicates that as the %ZnO increases, the OH% increases too. Then, at alkaline medium (pH 8), OH^−^ ions concentration increased, which resulted in the fast growth of ZnO to form a thick coating to the fabric.

### Zeta Potential and particle size distribution

The zeta potential is dependent on the surface charge which develops when ZnO is placed in a liquid. It is commonly used to predict and control dispersion stability. Figure 3(c) shows an average zeta potential of ZnO NPs extracted from the washing water for ZnO- coated cotton fabrics^49^. Zeta potential value gives information on to the stability of the synthesized nanoparticles. The value of zeta potential of the precipitated ZnO NPs after dilution is depicted from Fig. 3(c) to be 11.4 mV. Also, the value of the conductivity was estimated to be 1.06 mS/cm. The resulted values refer to the proper stability of ZnO NPs without noticeable aggregation.

Particle size distribution was measured by using ZS Malvern Zetasizer device coupled with an automatic titrator based on DLS and shown in Fig. 3(d). As shown in this figure, the size is polydisperse with three broad peaks centered at 82.4, 380.97, 5739.2 nm. This size is expected to be larger than the particle size that can be estimated from SEM image and XRD chart in Figs 1(c) and 2. This is because the aggregation of ZnO NPs in the aqueous solution in the case of DLS technique while no aggregation can occur in the case of SEM or XRD (the samples are measured in the dry state). Particle size obtained from DLS occurs in a solution form, so the nanoparticles may be swelled with time while measuring^50^.

### Water contact angle measurement

Wettability of the surface is an important property controlled by the chemical composition and geometrical structure of the surface^51,52^. So, it is significant to address the relationship between the structural and morphological properties of ZnO-coated fabrics and the water contact angle (WCA). The contact angle, θ_Y,_ for a liquid drop on a solid surface is determined by the surface free energies involved^18,53^:

2 $${{\boldsymbol{\gamma }}}_{{\bf{l}}{\bf{v}}}\,{\bf{c}}{\bf{o}}{\bf{s}}\,{{\rm{\theta }}}_{{\rm{Y}}}=({{\boldsymbol{\gamma }}}_{{\bf{s}}{\bf{v}}}-{{\boldsymbol{\gamma }}}_{{\bf{s}}{\bf{l}}})$$

where ${{\rm{\gamma }}}_{{\rm{lv}}}$, ${{\rm{\gamma }}}_{{\rm{sv}}}$, and ${{\rm{\gamma }}}_{{\rm{sl}}}$ are the (liquid/vapor), (solid/vapor), and (solid/liquid), tensions, respectively. The wettability of the cotton fabrics was examined by WCA measurements. The value of θ_Y_ was measured through sessile drop method. A 5 µl drop of deionized water was position on the surface of fabrics with micro-syringe. θ_Y_ was measured at four equidistant and images were recorded in each position was averaged.

**Figure 4:**
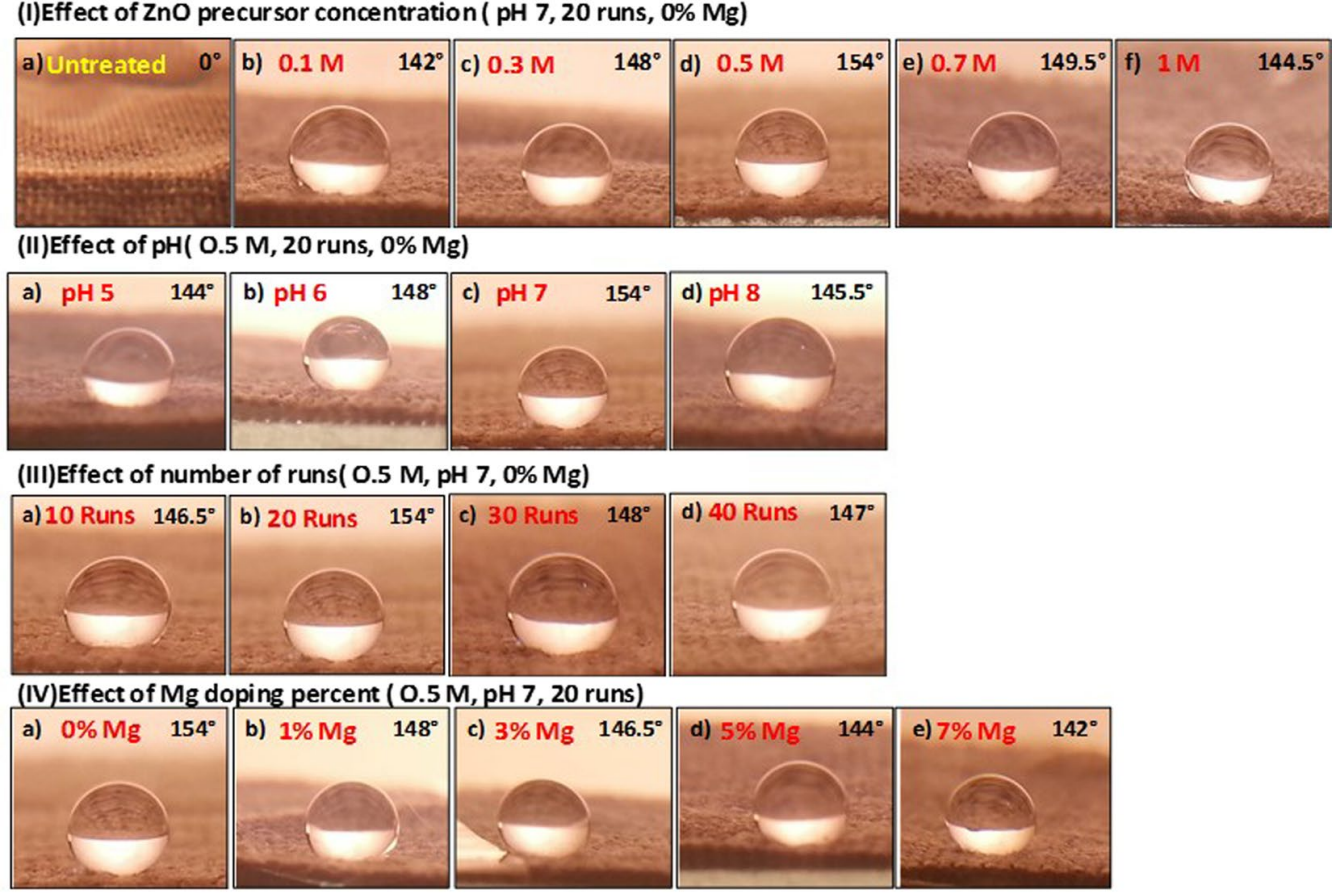
Contact angles photographs for ZnO- coated fabrics fabricated at different (I) ZnO precursor concentrations, (II) pH, (III) number of runs, and (IV) Mg doping percent.

Figure 4 shows the contact angle photographs of un-coated and ZnO- coated fabrics at different (I) ZnO precursor concentrations, (II) pH values, (III) number of coating runs, and (IV) Mg- doping percent. From these photographs, the WCAs have been evaluated, and the average values are shown in this figure. The natural cotton fabric can be completely wetted by water because of the abundance of hydroxyl groups in its molecular structure, and the static WCA is supposed to be 0°, as shown in Fig. 4(I) (a). It was observed that the different morphologies and structures of the ZnO-coated fabrics exhibit enhanced hydrophobic properties with θ_Y_ ≥ 142° as shown in Fig. 4. This refers to the increased surface roughness due to the ZnO coating.

**Figure 5:**
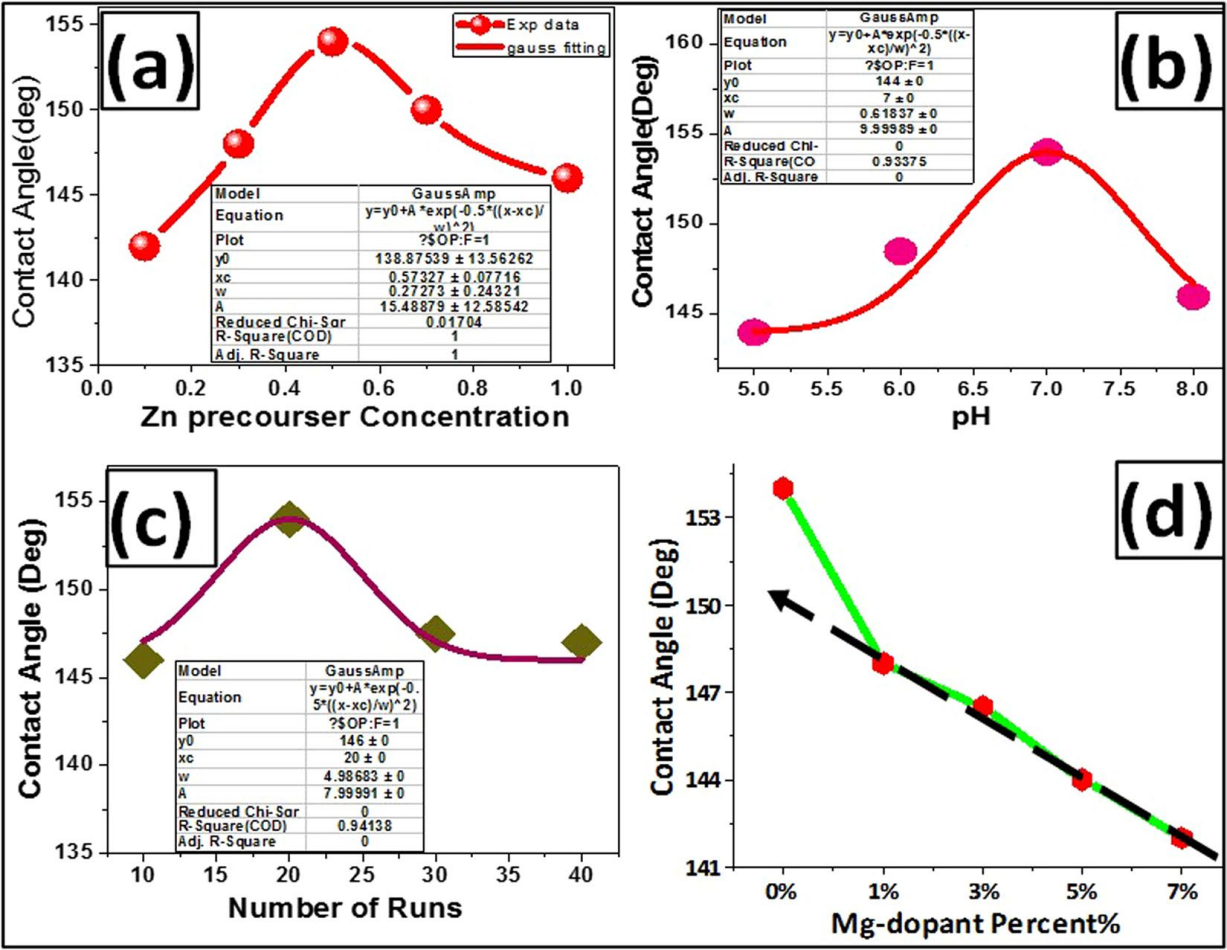
WCA of ZnO-coated fabrics versus (**a**) ZnO precursor concentrations, (**b**) pH values, (**c**) number of coating runs and (**d**) different Mg doping values.

The increase or decrease in the wettability is mainly due to the surface topography effect in which the grooves and air interfaces between the individual structures can minimize the water contact angle. It is previously well-known that the surface free energy and surface roughness are the measure of the surface wettability^18^. Such surface roughness is insufficient to form an increased hydrophobicity due to the high polarity of the surface that leads the water to fill the grooves quickly through the capillary action^18^. WCA (θ_Y_) is increased from 142^0^ to 154^0◦^ ± 1 as the precursor concentration increased from 0.1 M to 0.5 M and then decreased to 144.5^0^ with increasing precursor concentration to 1 M, Fig. 4(I). The drop of the contact angle at the low and high concentrations may be ascribed to the non-uniform coating at concentrations ≤0.3 M and the agglomeration of ZnO nanoparticles on the fabric surface at concentrations ≥0.7 M, as noted from SEM images. Also, the high dislocation density and preferential growth of ZnO along (0 0 2), which has the lowest surface free energy density (9.9 eV/nm^2^), can explain the enhanced hydrophobicity at the concentration 0.5 M. Figure 5(a) shows the variation of water contact angle with ZnO precursor concentration. The experimental data is well fitted with Gaussian equation; ${\rm{y}}=143+11.17\ast \exp (-17.3\ast {({\rm{x}}-0.525)}^{2})$.

Also, θ_Y_ is increased from 144^0^ to 154^0^ ± 1 as the pH value increased from 5 to 7 and then decreased to 145.5^0^ with increasing pH value to 8, Fig. 4(II). Thus, the structure and morphology of the coated ZnO at pH7 is the most promising for reaching the highly hydrophobic surfaces. This could be ascribed to the microstructural and crystallinity changes that happen by tuning the pH. The preferred growth along (002) direction realized in ZnO –coated fabrics significantly reduced at pH 8. Then, the crystallinity deterioration leads to increase of the surface roughness and free energy at higher pH value and hence reducing WCA. Similar results were reported for ZnO nanodisks and nanorods^18^. The variation of water contact angle with pH value is presented in Fig. 5(b), and the fitting equation is y = 144.3 + 9.5 ∗ exp (−1.8 × (X − 7)^2^).

With increasing the number of coating runs to 20 runs, the contact angle increased till reached its maximum value (154^0^) as shown in Fig. 4(III). Above 20 runs, the contact angle decreased and reached 147^0^ at 40 runs. From Table 1, the size of the ZnO crystallites decreased, and the dislocations density increased significantly when the number of runs increased to 40 runs. As a result, the surface roughness is affected, and the contact angle is decreased^54^. Also, the agglomeration of ZnO nanoparticles as noted from SEM images may be another important reason for the decline of the contact angle. The values of the contact angle versus the number of runs are illustrated in Fig. 5(c) and fitted with the Gaussian equation; y = 147 + 7 * exp (−23.9 ∗ 10^−3^ ∗ (X − 21.1)^2^). Then, the superhydrophobic ZnO-coated fiber can be achieved by optimizing the ZnO precursor concentration at 0.5 M, pH value at 7, and the number of coating runs to 20 runs.

From Fig. 4(IV), the contact angle decreased from 154^0^ to 142^0^ by increasing the Mg doping percent from 0% to 7% Mg. The experimental data decreased linearly according to the relation y = 15 − 0.12× by increasing the Mg-doping percent from 1 to 7% as shown in Fig. 5(d). Based on the study of the structural properties, the preferred orientation of ZnO along (0 0 2) direction on the fabrics was significantly decreased with increasing Mg-doping percent. This is because the crystallinity deterioration which leads to increase of the surface free energy. As a result, the WCA decreases linearly by increasing the Mg-doping percent.

### The environmental durability and the abrasion resistance of ZnO-coated fabric

**Figure 6:**
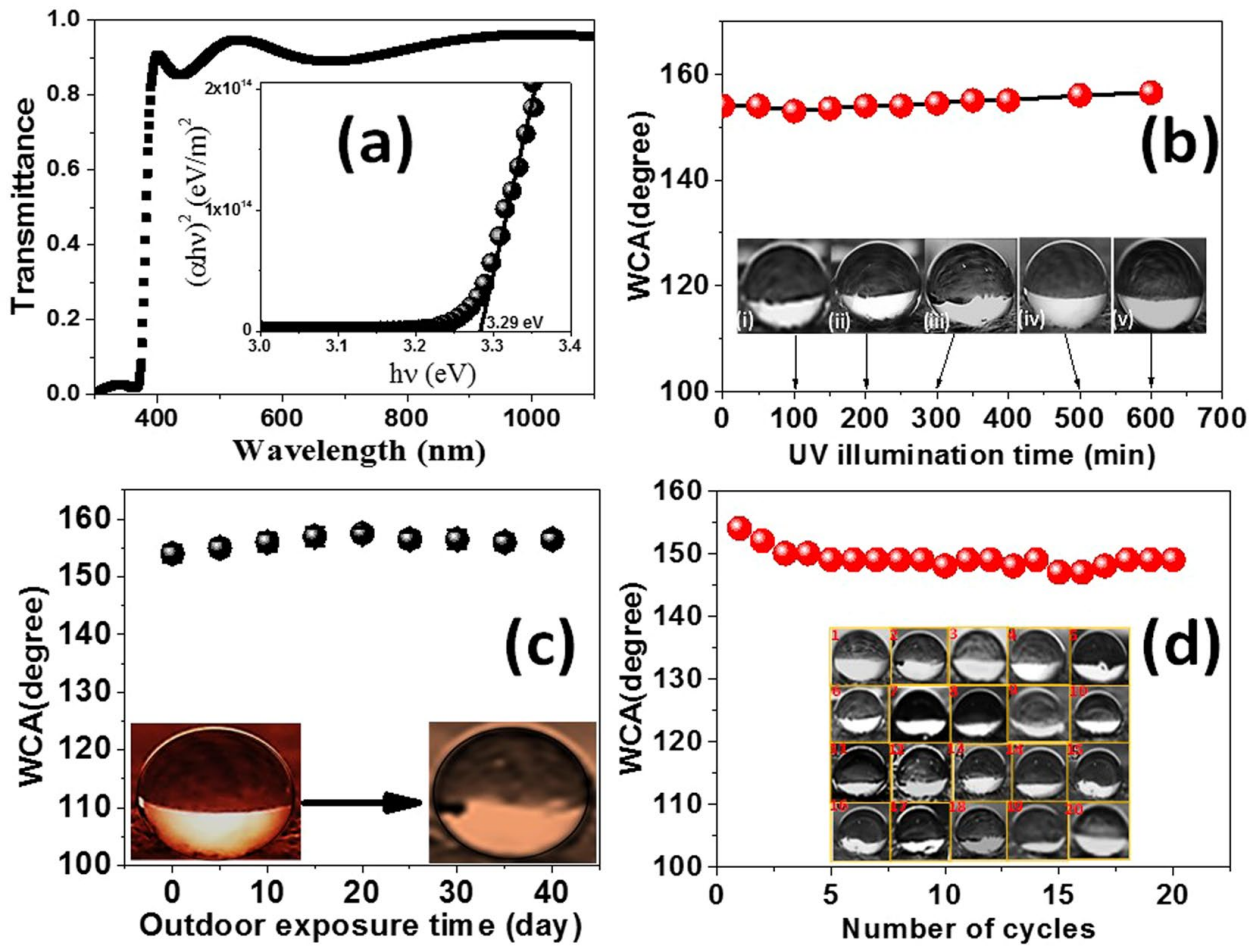
Transmittance spectrum of the optimized ZnO-coated fabric. Inset: plotting of (αhν)^2^ versus hv for the optical band gap (*E*_*g*_) calculation. The variation of WCA versus (**b**) UV illumination time, (**c**) Outdoor exposure time, and (**d**) number of polishing cycles for the optimized ZnO-coated fabric. Insets: the photographs of the water droplets on the optimized ZnO-coated fabric.

The optical transmittance (T) spectrum, Fig. 6(a), of ZnO deposited at the optimized conditions was measured and corrected for the substrate effect^55^. The ZnO coating showed a high transmittance in the visible region accompanied with sharp fundamental absorption band edge near to *λ* = 375 nm. This high transmittance is attributed to small scattering effects resulting from the structural homogeneity of the deposited ZnO and the apparent high crystallinity (Figs 1c, S1c, and Fig. 2). Continuously oscillating maxima and minima (interference pattern) at different wavelengths suggest the optical homogeneity of deposited ZnO. From the transmittance spectrum, the optical band gap (*E*_*g*_) was calculated by plotting (*αhν*)^2^ versus *hv*^40,42^, as shown in the inset of Fig. 6(a). The value of *E*_*g*_ was obtained to be 3.29 eV by extrapolating the linear part to *α* = 0.

So, it is important to study the effect of UV illumination on the contact angle of the optimized ZnO-coated fabrics. To carried out this test, four mercury lamps (8 W, wavelength = 265 nm) were used as the UV illumination source^56^. The UV source was positioned at 10 cm from the sample. Figure 6(b) shows WCAs of the optimized ZnO-coated fabric versus UV illumination time. The inset shows the photographs of the water droplets on the optimized ZnO-coated fabric at different UV illumination time. The positioned water droplets on the optimized ZnO-coated fabric possessed near-spherical shapes. The water contact angle of the optimized ZnO-coated fabric remained almost unchanged after 700 min UV illumination or slightly increased to 156.5° ± 1.5. Also, water droplet positioned on the optimized ZnO-coated fabric illustrated near-spherical shapes for over 60 min. These results are suggesting stable and durable superhydrophobicity. Because the optimized ZnO-coated fabric showed UV-durable superhydrophobicity, this optimized fabric might have possible applications in outdoor uses. Similar results were reported by Wang *et al*. for flower-like microstructure ZnO-coated mesh films^56^. Figure 6(c) shows the effect of the outdoor time on the WCAs of the optimized ZnO-coated fabric. The inset illustrates the water droplets on the optimized ZnO-coated fabric before and after outdoor exposed for 20 days. The water droplets possessed almost spherical shapes after 20 days’ outdoor exposure time as shown in the inset. The optimized ZnO-coated fabric kept WCA ≥ 154° in an outdoor environment. Therefore, the optimized ZnO-coated fabric exhibited long-term superhydrophobic stability under UV illumination and in the outdoor environment.

The physical abrasion is another challenge to artificial superhydrophobic surfaces, which will not only destroy the micro/nanostructures but also reduce the hydrophobic property from the top layer of the hydrophobic surfaces^57^. To evaluate the influence of mechanical abrasion in our optimized sample, the abrasion resistance of the optimized superhydrophobic ZnO-coated fabric was conducted by dragging a piece of 1500-mesh sandpaper of area 2.5 × 2.5 cm^2^ under a weight of 1 kg in one direction^57,58^. The sandpaper was dragged at a speed of 1 cm/s. The WCA as a function of the number of polishing cycles is shown in Fig. 6(d). The inset of Fig. 6(d) shows the photographs of the water droplets on the optimized ZnO-coated fabric after each of the polishing cycles. The water contact angle slightly decreased as shown in Fig. 6(d). Hence, this ZnO-coating shows remarkable mechanical durability and excellent adhesion to the fabric even after 20 cycles by keeping its superhydrophobicity with WCA ≥ 149°. Figure 6(c,d) verifies that the optimized ZnO-coated fabric has excellent abrasion resistance and environmental durability. Thus, it could find more specific indoor or outdoor applications.

### Antibacterial properties

**Figure 7:**
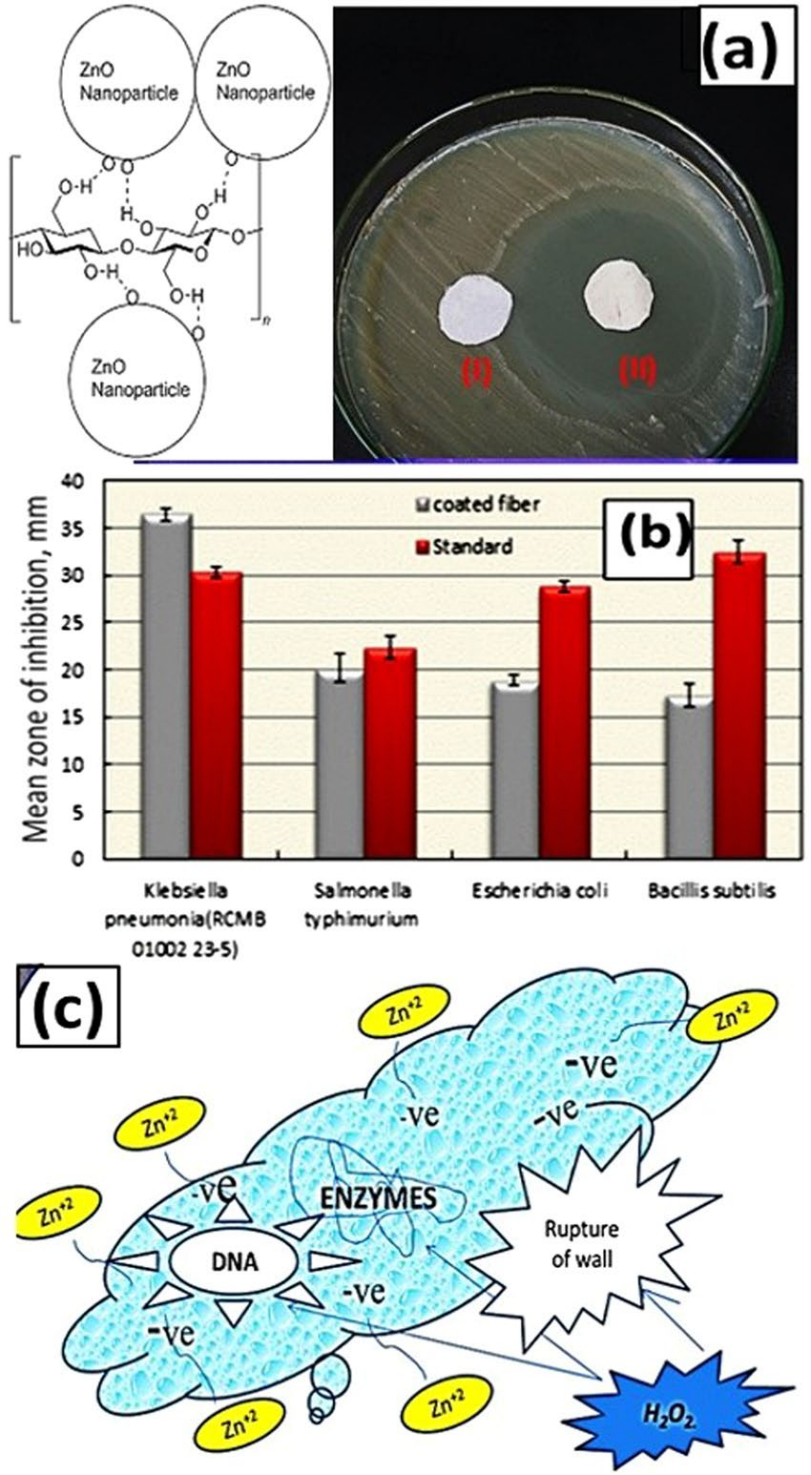
(**a**) Antibacterial activity of (**a**) uncoated cotton fabric and (**b**) ZnO-coated fabric against Klebsiella pneumonia. The inset is the structural formula of ZnO-coated cellulosic cotton fabric, (**b**) effect of ZnO-coated fabric on zones of growth inhibition (mm) of different species of bacteria, and (**c**) schematic diagram of the different antibacterial mechanisms of ZnO-coated fiber.

Based on the improved self -cleaning properties, ZnO- coated fabrics could be involved in biological self-cleaning and biomedical applications. The antibacterial activities of the uncoated cotton fabric and the optimized ZnO-coated fabric were investigated against various types of gram-positive and gram-negative bacteria using diffusion agar method. The results for the gram-positive bacteria (*B. subtilis*) and gram-negative bacteria (*K. pneumonia*, *E. coli*, and *S. typhimurium*) were tabulated in Table 2 and shown in Fig. 7.

**Table 2:** Antibacterial activity of uncoated cotton fabric and optimized ZnO-coated fabric.

Tested Microorganisms	Un-coated Fabric	ZnO-coated Fabric	ST.(30 µg)
**Gram-Positive Bacteria:**			*Ampicillin*
*B. subtilis* (RCMB 000107)	7.3 ± 0.58	17.3 ± 1.2	32.4 ± 1.2
**Gram-negative Bacteria:**			*Gentamicin*
*K. pneumonia* (*RCMB 01002 23-5*)	NA	36.4 ± 0.58	30.3 ± 0.63
*E. coli* (RCMB 010052)	NA	18.8 ± 0.58	28.8 ± 0.58
*S. typhimurium* (RCMB 010072)	10.3 ± 0.63	20.1 ± 1.5	22.3 ± 1.2

Mean zone of inhibition in mm ± Standard deviation beyond well diameter (6 mm) produced on a range of environmental and clinically pathogenic microorganisms using (20 mg/mL) concentration of tested samples. 10^5^ CFU/mL was utilized for antibacterial assay.

Ampicillin and Gentamycin were used as reference antibiotics. Figure 7(a) illustrates the difference between the antibacterial activity of (I) uncoated fabric and (II) ZnO-coated fabric against *K. pneumonia*. It can be observed that the uncoated fabric has no activity against *K. pneumonia*, whereas ZnO-coated fabric has very high activity. Also, ZnO- coated fabrics *in-vitro* highly antimicrobial activity against all the tested microorganisms as shown in Fig. 7(b). These improved antibacterial activities might be attributed to different mechanisms as displayed in Fig. 7(c). The reaction of ZnO NPs with the cell of bacteria and liaison of them to bacteria cell wall causing killing of bacteria by rupture of their cell wall^59^. Also, the release of Zn^2+^ with zeta potential = 11.4 mV disturbs the metabolic process of bacteria such as glycolysis, trans-membrane proton translocation and acid tolerance by the electrostatic attraction with the negatively charged polysaccharides of lipopolysaccharide, which predominate over the amide^60,61^. Also, the reaction of ZnO with water molecules leads to the production of H_2_O_2_^.^, OH ions, and other ROS by non-photocatalytic processes. These species can penetrate the cell wall and damage DNA and enzymes of bacteria^62^. Moreover, ZnO surface defects and surface charges can contribute to the antibacterial activity of ZnO-coated fiber as its surface has many edges and corners with potential reactive surface sites^50,51^. It is known that gram-positive bacteria (e.g., *B. subtilis*) have a steeper defense system as compared to gram-negative bacteria (e.g*., K. pneumoniae*)^63,64^. Gram-positive bacteria have a thick peptidoglycan cell wall, whereas the cell wall of the gram-negative bacteria is mostly made of tightly packed lipopolysaccharides (LPS) that offer less efficient protection against the penetration of toxic species in the cytoplasm^64^. As a result, the ZnO- coated fiber is more active against the gram-negative bacteria than against gram-positive bacteria, Fig. 7(b). The negative charge on *K. pneumoniae* cell wall is higher than that on *B. subtilis* cell wall^65^. This leads to intense electrostatic interaction on *K. pneumoniae* bacterial surface with Zn ions^66^, which in turn blocks or inhibits the growth of *K. pneumoniae* in lag phase itself. This reveals that optimized ZnO coating is significantly active against *K. pneumoniae* in the lag phase.

Actually, the antimicrobial activity of a tested catalyst should be compared to that of the standard antibiotic that practically used against certain bacteria. Different bacterial responses toward ZnO catalyst within –ve gram species may be attributed to the relative bacterial uptake of the antimicrobial agent during overall bacterial growth phases.

In contrast to previous works^67–73^, our findings achieved a dual effect on the fabrics; superhydrophobic and antibacterial effect. ZnO-coated fabrics showed hydrophobic properties with super contact angle (154^0^) at the same time exhibited strong antimicrobial activities against various types of bacteria. To date, a limited number of papers have been reported about using nanostructures materials in the preparation of different types of self-cleaning. Table 3 illustrated the comparison between some coated fabrics and the prepared ZnO-coated fabric in this study. Superhydrophobic ZnO nanocoating under the optimized conditions can enhance the self-cleaning and antibacterial activities of the cotton fabrics.

**Table 3:** Comparison between metal oxide coated fibers for different applications.

Type of coating on fiber	Type of self -cleaning	Efficiency of coating for self-cleaning	Ref.
ZnO coated fiber by pad –dry –cure method	Biological self-cleaning(antibacterial) only	- showed 99.9 and 80% reduction of *S. aureus* and *E. coli*.	^67^
Polyester fiber coated ZnO	Biological self-cleaning only	*- presented a moderated activity against S. aureus* and *K. pneumonia*.	^68^
cotton fabrics coated with TiO_2_, Perfluorodecyl trichlorosilane (PFTDS) and stearic acid by sol-gel	Physical self-cleaning only	- showed super-hydrophobic cotton fabrics with contact angle ranged from 94 to 163^0^.	^69^
Polyester fabrics coated with TiO2	Chemical and biological self-cleaning	- eliminated the chromophores of the Acid Blue 113 stain under UV and daylight irradiation. - showed excellent antibacterial activity against *E. coli* only.	^70^
cotton fabric coated with zinc oxide	Biological and physical self-cleaning	-reduced the *E. coli* growth only and the contact angle increased to 104^°^.	^71^
cotton fibers coated with zinc oxide by Ultrasonic irradiation	biological self-cleaning only	-showed a great drop in the bacteria activity against *E. coli* and *S. aureus*	^72^
cotton fabrics exploiting zinc oxide by dip-pad-dry-cure	Physical self -cleaning	-exhibited good photocatalytic activity against Methylene Blue and reached 100% after 24 h. -showed excellent ultraviolet blocking properties.	^73^
Cotton fiber coated with zinc oxide by sol-gel	Physical self-cleaning and biological self –cleaning	-exhibited enhanced antibacterial activities against different species of bacteria, especially K. pneumonia. - showed superhydrophobic water contact angle (154°) at the optimum conditions.	Present paper

The economic importance of the introduced self-cleaning and antimicrobial textiles can be summarized as follows: ease of preparation and environmental protection, lowering the consumption of chemicals (such as detergents and dry-cleaning solvents), water, and energy; and offering a sterile textile with low-cost that can be used in wards and ICU.

## Conclusion

Cotton fabric coated with ZnO NPs has been successfully prepared via a facile and cheap sol-gel spin coating technique. The uniform coating of the cotton fabrics with ZnO NPs was confirmed by FTIR, Zeta potential, XRD, and SEM. The conditions of ZnO-coating such as the precursor ZnO concentration, pH value, the number of coating runs, and Mg doping percent were optimized. The relationship between the structural and morphological properties of ZnO-coated fabrics and the water contact angle (WCA) was investigated. The superhydrophobic ZnO-coated fiber (154°) can be achieved by optimizing the ZnO precursor concentration at 0.5 M, pH value at 7, and the number of coating runs to 20 runs. The optimized ZnO-coated fabric has excellent abrasion resistance and environmental durability under UV illumination and in an outdoor environment. The antibacterial activities of the optimized sample were studied using some species of gram-positive and gram-negative bacteria. The functionalized fiber with ZnO NPs exhibited enhanced antibacterial activities against different species of bacteria, especially *K. pneumonia*. Therefore, the coating of textile with ZnO NPs at the optimized conditions open up the possibility of multifunctional finishing of textiles with a single treatment process. The significant advantages of the presented technology; low fabrication cost, better performance regarding hydrophobicity and antibacterial activity, and large-scale fabrication availability; suggest the use of this technology for the biochemical and biomedical application.

## Methods

### Fabrics pre-treatment

The cotton fabric was washed firstly with deionized water, acetone, and ethanol to remove any impurities. All washing processes were carried out under ultrasonication at 80 °C for 10 min.

### Preparation of ZnO sol

Zinc acetate dihydrate (Zn(CH_3_COO)_2_.2H_2_O), 2-methoxy ethanol (C_3_H_8_O_2_) and monoethanolamine (C_2_H_7_NO, MEA) were used as a precursor, solvent, and stabilizer, respectively. Different concentrations of zinc acetate dihydrate (0.1, 0.3, 0.5, 0.7 and 1 M) were dissolved in 2-methoxy ethanol for 10 min, and then MEA was added drop wisely to the solution. The molar ratio of MEA/zinc acetate = 1. The pH of 0.5 M prepared solution was controlled by adding sodium hydroxide (NaOH) or acetic acid (CH_3_COOH). The pH value was adjusted at 5, 6, 7 and 8. Magnesium acetate tetrahydrate (Mg(CH_3_COO).4H_2_O was used as the dopant material. Magnesium acetate in the required stoichiometry (1%, 3%, 5% and 7% doping percent) was slowly added into 0.5 M Zn acetate precursor solution with a pH of 7. The resultant solutions were stirred at 60 °C for 2 h to yield a homogeneous and stable colloid solution. The prepared sols were aged for 24 h at room temperature.

### Coating of cotton fabrics with ZnO nanoparticles

The prepared solutions at different conditions were coated onto cotton fabrics by a spin coater at the rate of 1100 rpm for 60 s. Subsequently, the coated fabrics were heated for 10 min to remove the residual solvent. The procedures from coating to heating were repeated 20 times. The number of runs for the sample with optimum conditions (0.5 M precursor concentration, pH 7 and 0%Mg doping percent) was changed from 10 to 40 runs. Then, the whole coated fabrics and a part of the sol prepared at optimum conditions were annealed in a furnace at 150 °C for 2 h.

## Samples characterization

### Structures and morphologies characterization

The crystalline structures of ZnO nanoparticles and ZnO-coated fabrics were determined using high-resolution X-ray diffractometer system (model: PANalytical X’Pert Pro, Holland) with CuK α radiation (${\rm{\lambda }}$ = 1.5406 A°) operated at 40 kV and 35 mA. FTIR spectra were recorded using a Bruker spectrometer (Vertex 70 FTIR-FT Raman). The morphologies of the samples were obtained using scanning electron microscopy (SEM, model: Quanta 250 with Field Emission Gun) attached with energy dispersive X-ray (EDX) unit.

### Zeta potential and particle Size distribution

The stability of the suspensions was monitored in water. Zeta potential measurements were carried out using a Zetasizer Nano ZS instrument with MPT-2 automated titrator (Malvern Instruments Ltd., Worcestershire, UK) in 10 mM NaCl. This solution is convenient for zeta potential determination because of its optimal ionic strength. Dynamic light scattering (PCS-Photon Correlation Spectroscopy) was applied to measure the hydrodynamic diameter of the particles in ultrapure water. The scattering was measured at an angle of 173° to the incident He/Ne laser beam. The autocorrelation functions carried out using the Contin algorithm to derive intensity-weighed particle size distributions. A second-order cumulant analysis of the correlation function and application of the Stokes-Einstein relation, taking the viscosity of the suspension into account, resulted in the intensity-weighted average hydrodynamic diameter. The ZnO NPs-coated cotton fabrics were washed with distilled water several times to remove the weakly adsorbed ZnO NPs on the surface of the fabrics. Then the solution was centrifuged for 1 h at 4500 rpm to precipitate the unreacted ZnO NPs. The resultant white powder was then washed with distilled water and dried at 160 °C for 120 min. The obtained washing water that contains the residual ZnO NPs was centrifuged at 4500 rpm for 1 h. The dried powder was dilute with distilled water, and zeta potential was measured to confirm the successful formation of ZnO NPs on the surface of fabrics.

### Surface wettability measurements

The surface wettability of the different samples was characterized via measuring water contact angle, WCA (Θ), by the sessile drop method utilizing a CAM 200 Optical Contact Angle Meter (KSV Instruments). A 5 µl droplet of distilled deionized water was positioned on the surface via a micropipette and images were captured to measure the angle formed at the liquid/solid interface. All the contact angles were determined by averaging values measured at 5–6 different points on each sample surface.

### Antibacterial activities characterization

Typically, the antibacterial activity was tested for both untreated cotton fabric and ZnO- coated fabric at the optimum conditions^74^. The antibacterial activity of both treated and the untreated cotton fabric was tested against some species of Gram-positive and Gram-negative bacteria using modified agar diffusion assay (disc test) maintained at 37 °C for one day during the incubation period. The Gram-positive bacteria are *S. aureus*, *B. subtilis*, *E. faecalis*, and *B. cereus*. The Gram-negative bacteria are *K. pneumonia, P. aeruginosa*, *E. coli*, and *S. typhimurium*. The required nutrient agar medium was prepared by mixing peptone (5 g), beef extract (3 g) and sodium chloride (5 g) in 1000 ml distilled water. pH value was adjusted at 7. Finally, agar (15 g) was added to the solution. Sterilization of agar medium was done in a conical flask at a pressure of 6.8 kg (15 lbs) for 30 min. The standard conditions of 10^6^ CFU/mL (Colony Forming Unit/mL), then 10^5^ CFU/mL were utilized for antibacterial assay. This medium was transferred into sterilized Petri dishes. The bacteria species (50 µl) cultures were spread on the solid surface of the media after solidification of the media. Over this inoculated petri dish, small pieces of untreated fabric and the treated cotton fibers at optimum conditions were distributed and incubated for two days at 37 °C in an incubation chamber to observe the inhibition zone. All the experiments were performed in triplicate to confirm their reproducibility. Using SPSS version 21, Means and standard deviation (SD) values were computed and P values less than 0.05 were considered as statistically significant.

## Electronic supplementary material

Supplementary Information
