# Supplementary material for: Production and Characterization of Superhydrophobic and Antibacterial Coated Fabrics Utilizing ZnO Nanocatalyst
Source: Sci Rep. 2018 Mar 2;8:3925. doi: 10.1038/s41598-018-22324-7 (PMC5834644; doi:10.1038/s41598-018-22324-7)
Supplement: Supplementary file 1 — Supplementary Information [file 41598_2018_22324_MOESM1_ESM.pdf]

Supplementary data

Production and Characterization of Superhydrophobic and Antibacterial Coated Fabrics Utilizing ZnO Nanocatalyst

Mohamed Shaban[1,*], Fatma Mohamed[1,2], Semsem Abdallah[1,2]

[1] Nanophotonics and Applications (NPA) Lab, Department of Physics, Faculty of Science, Beni - Suef University, Beni-Suef, 62514, Egypt

[2] Department of Chemistry, Faculty of Science, Beni-Suef University, Beni-Suef 62111, Egypt

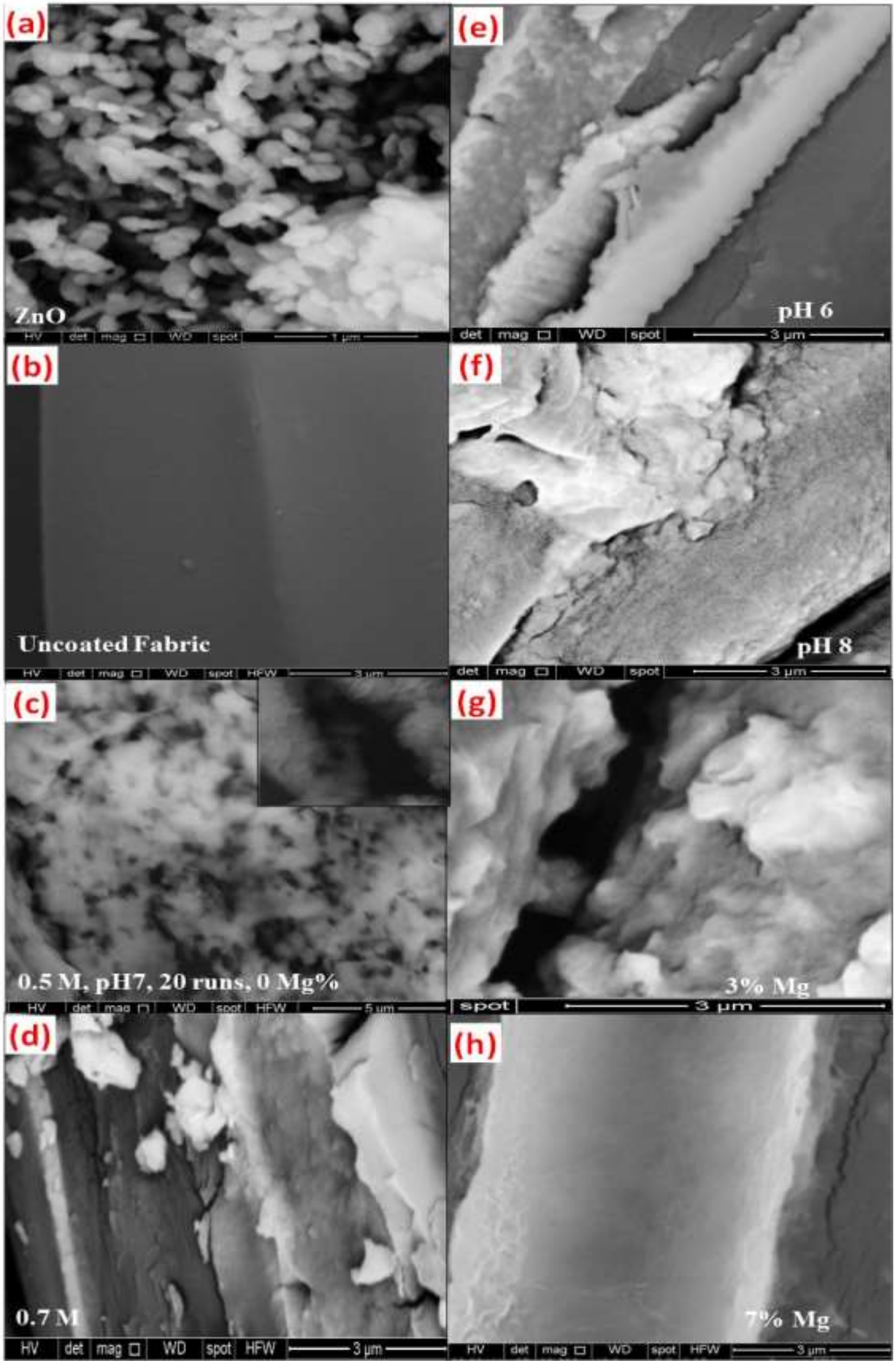

(a)
ZnO
1 µm
(b)
Uncoated Fabric
3 µm
(c)
0.5 M, pH7, 20 runs, 0 Mg%
5 µm
(d)
0.7 M
3 µm
(e)
pH 6
3 µm
(f)
pH 8
3 µm
(g)
3% Mg
3 µm
(h)
7% Mg
3 µm

Figure S1. **SEM images of (a) ZnO nanoparticles;(b) untreated cotton fabric; ZnO coated fabric at different concentrations of ZnO precursor solution: (c) 0.5 M and (d) 0.7 M at pH 7 and 20 runs; at different pH values: (e) pH 6 and (f) pH 8 at 0.5M precursor concentration and20 runs; at different Mg doping percent: (g) 3% Mg and (h) 7% Mg at 0.5M precursor concentration, pH 7 and 20 runs.**
